# Alterations in Synthesis and Repair of DNA during the Development of Loach *Misgurnus fossilis*

**DOI:** 10.3390/jdb4010006

**Published:** 2016-01-27

**Authors:** Leonid V. Gening, Andrei V. Lakhin, Irina V. Makarova, Valentina V. Nenasheva, Ludmila E. Andreeva, Vyacheslav Z. Tarantul

**Affiliations:** Institute of Molecular Genetics, Russian Academy of Sciences, 2 Kurchatov Square, 123182 Moscow, Russia; geni@img.ras.ru (L.V.G.); lahin9@mail.ru (A.V.L.); ivmakarova@img.ras.ru (I.V.M.); val-nenasheva@mail.ru (V.V.N.); leandr@img.ras.ru (L.E.A.)

**Keywords:** DNA polymerase iota, loach, DNA synthesis, DNA repair

## Abstract

Using a modified radiolabeled primer extension method (we named this modification misGvA—“misincorporation of G *versus* A”) we have investigated the DNA synthesis and repair at early and late stages of development of loach *Misgurnus fossilis.* The misincorporation activity of DNA polymerase iota (Pol ι) in wild-type loach could not be detected by this method at any stage of loach development. In transgenic loach overexpressing human Pol ι we have shown that the bypassing of DNA synthesis arrest after incorporation of mismatched nucleotide by Pol ι (the T-stop) was not associated with this enzyme. Non-transgenic loach larvae are virtually lacking the capacity for error correction of DNA duplex containing a mismatched nucleotide. Such repair activity develops only in the adult fish. It appears that the initial stages of development are characterized by more intensive DNA synthesis, while in terminal stages the repair activities become more prominent. The misGvA approach clearly indicates substantial changes in the DNA synthesis intensity, although the role of particular replicative and repair DNA polymerases in this process requires further study.

## 1. Introduction

The DNA of all living organisms is constantly exposed to disturbing factors both exogenous and endogenous in nature [1,2]. It has been estimated that roughly 10^3^–10^6^ DNA lesions occur in each cell daily [3,4]. In that context, an effective DNA repair system is indispensable for normal activity of cells of any type of organism. To avoid the deleterious consequence of a stalled replication fork, cells use specialized DNA polymerases to traverse the damage. This process, termed “translesion DNA synthesis” (TLS), gives the cell additional time to repair the damage before the replicase returns to complete genome duplication. In many cases, this damage-tolerance mechanism is error-prone, and cell survival is often associated with an increased risk of mutagenesis and carcinogenesis. 

Most DNA repair mechanisms were studied in differentiated cells, in which DNA synthesis proceeds only during a small proportion of the cell cycle, and can be temporally arrested for the repair of damaged DNA sequences [5,6,7]. At the same time, it was shown that the repair mechanisms are considerably different, even between proliferating and postmitotic cells in adult organisms [8,9]. In this regard, it can be expected that the mechanisms of DNA synthesis and repair in the quickly dividing cells at early developmental stages might also differ from those observed in the cells of an adult organism. To a certain degree this hypothesis relies on the fact that many processes of DNA repair are characterized by a slow rate since they are quite complex and proceed through multiple steps [10,11]. Consequently, the fast division of cells in embryos can exclude the contribution of at least some of these repair processes.

One of the main sources of mutations in cellular DNA might be conditionally-provoked by the misincorporating activity of one or multiple DNA polymerases [12,13]. The decrease of overall fidelity in DNA synthesis might be caused by the presence of increased concentrations of bivalent metal cations like Cd^2+^ and Mn^2+^ [12,14,15].

DNA polymerase iota (Pol ι) participates in various types of translesion DNA synthesis and is the most error-prone polymerase among repair DNA polymerases [16,17]. This enzyme is characterized by strong activation of error-prone DNA synthesis in the presence of Mn^2+^ ions [18,19]. We have previously demonstrated that the highest Pol ι activity is observed in the tissues of mouse embryos, compared to newborn and adult mice [20]. This points towards possible special roles for Pol ι in DNA synthesis and repair in the cells of developing organisms. At the same time, the role of Pol ι in these processes remains largely obscure and requires further investigation. In the current paper, we have assayed the changes in DNA synthesis and the repair of DNA synthesis products bearing misincorporated nucleotides in cell extracts of loach larvae in comparison with intact adult fish.

## 2. Materials and Methods

### 2.1. Generation of Transgenic Loach Larvae Bearing Gene Encoding DNA Polymerase Iota 

DNA fragments corresponding to human Pol ι cDNA along with 60 bp 5’-untranslated region sequence were inserted between the NcoI and BamHI sites of pEGFP-C1 vector (Clontech, Montain View, CA, USA), downstream of a cytomegalovirus (CMV) promoter, to generate the fusion protein eGFP-POLI (pEGFP-POLI vector), as described previously [21]. The plasmid containing cDNA encoding an inactive mutant form of the Pol ι protein (D126A-E127A) fused with eGFP was generated using site-specific mutagenesis of pEGFP-POLI vector by Evrogen (Moscow, Russia). To obtain transgenic 5–10 day loach larvae *(Misgurnus fossilis L),* the linearized recombinant DNAs encoding active or inactive forms of human Pol ι were injected into fertilized eggs at the germinal disc stage, as was previously described [21]. The expression of Pol ι in loach larvae was detected by Western blotting, and the activity of Pol ι was determined by primer extension reaction (the procedures and figures were presented in our previous work [21]). 

### 2.2. Preparation of Cellular Extracts for DNA Polymerase Reaction

The animals were treated in accordance with the European Society Council 86/609/EEC requirement concerning the use of animals for experimental studies. The extracts of mouse testis and brain were obtained from 2-month old males from the lines 129 and C57B1. For loach, the extracts were obtained from 7-day larvae (10 in each experiment), and from the testis and brain of adult fish (one per experiment).

Tissues were homogenized on ice using a Teflon homogenizer and glass beads in the extraction buffer containing 10% glycerol, 1% DMSO, 0.5% Tween 20, 2.5 mM DTT, 1 mM PMSF in 1× PBS (pH 7.4). The volume of extraction buffer taken for each sample was calculated by following proportion: 2 µL of extraction buffer were used per 1 mg of homogenized tissue. The obtained homogenate was centrifuged at 4°C, 14,000 *g*, for 10 min. The supernatant was used directly as the enzyme preparation. Protein concentration was measured with Protein Assay reagent (BioRad, Hercules, CA, USA) and diluted to 5 mg of protein per 1 mL of extract. 

### 2.3. Substrates for DNA Polymerase Reaction

As the substrate No. 1 for detecting Pol ι activity in cell extracts we have utilized two complementary oligodeoxyribonucleotides: a 17-mer primer 5’-GGAAGAAGAAGTATGTT-3’ and 30-mer template 5′-CCTTCGTCATTCTAACATACTTCTTCTTCC-3′, that, after annealing, form a duplex with an overhanging 5′-end. The studies of reparation processes were performed using substrate No. 2, which included 18-mer primer 5’-GGAAGAAGAAGTATGTTG-3’ and the same 30-mer template that was present in substrate No. 1. Thus, substrate No. 2 contains a non-complementary nucleotide at the 3’-end of the 18-mer primer and corresponds to an aberrant product synthesized by Pol ι on substrate No. 1.

Labeling of the primers at the 5’-end was performed using 10 units of T4 phage polynucleotide kinase (PNK) and 2 MBq [γ-^32^P] ATP in 70 mM Tris-HCl buffer (pH 7.6), that included 10 mM MgCl_2_ and 5 mM DTT, during 30 min at 37 °C. The polynucleotide kinase was then inactivated by incubation at 70 °C for 10 min. The substrate for the enzymatic reaction was obtained after annealing 2.7 µM of the labeled primer with 4 µM of template in PNC buffer supplemented with 100 mM NaCl at 73 °C for 3 min, followed by cooling to room temperature.

### 2.4. Radiolabeled Primer Extension Reaction

The DNA synthesis reaction with substrates No. 1 and No. 2 was performed in 20 µL of reaction mix, containing 25 nM of substrate with labeled primer, along with 50 mM Tris-HCl (pH 8.0), 0.25 mM MnCl_2_, 0.5 mM of both dATP and dGTP (in the presence of all four dNTPs the efficiency of the Pol ι activity detection decreases), and 4 µL of extract of the corresponding sample. Basal level of DNA synthesis was achieved under a near-physiological concentration of Mg^2+^ (250 µМ) [22,23]. The incubation of the reaction mixture with tissue extracts at 37 °C was performed for 10 min. The reaction was stopped, the reaction products were separated by electrophoresis, and a radio-autograph was obtained as described earlier [24]. The data was analyzed with Image Quant software. In all cases, quantitative parameters were determined from results of three to five independent experiments. 

## 3. Results and Discussion

### 3.1. Human Pol ι Overexpression-Induced Alterations in DNA Synthesis in Tissue Extracts of Loach Larvae 

**Figure 1:**
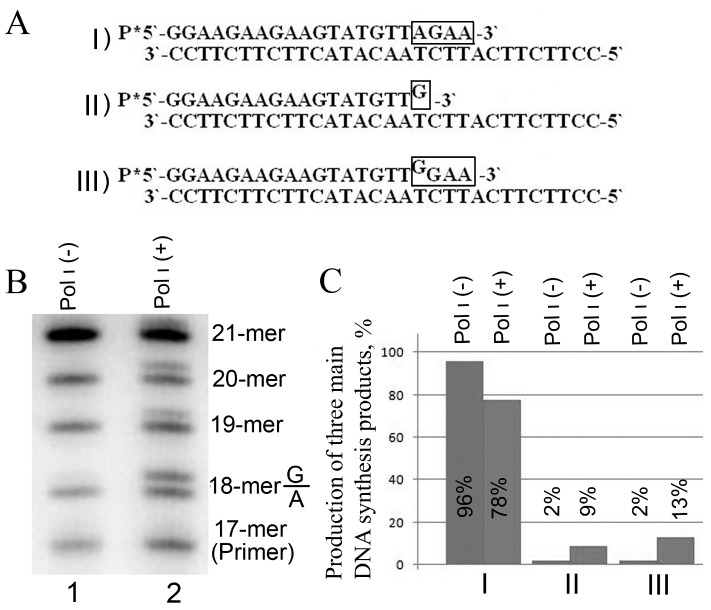
DNA synthesis in extracts of transgenic loach larvae expressing human Pol ι. In all cases, quantitative parameters were determined from the results of three to five independent experiments. (**A**) Alternative DNA synthesis products, generated in vertebrate cell extracts with substrate No. 1. The frame highlights the nucleotides that are incorporated into substrate No. 1 during DNA synthesis; (**B**) Electropherogram of DNA synthesis products using substrate No. 1, generated in cell extracts of transgenic loach larvae, that either express inactive (lane 1) or active form of Pol ι (lane 2). The products incorporating mismatched G that results from Pol ι activity correspond to bands that migrate more slowly in gel; (**C**) Histogram representing total main DNA synthesis products (depicted in (A)), generated in extracts of transgenic loach larvae expressing non-functional and active forms of Pol ι (the data was derived from electropherogram (B) and calculations were performed using ImageQuant software). Standard deviation in all experiments was within 5%.

We have previously demonstrated that the misincorporation activity of Pol ι in intact loach could not be detected at any stage of development [21]. After injection of human Pol ι encoding gene into loach embryos, we have observed the appearance of activity of this enzyme in cellular extracts of GFP-expressing larvae (Figure 1B) by using the radiolabeled primer extension method [25,26]. The primer extension reaction was performed with substrate No. 1 in reaction medium containing 250 µM of Mg^2+^ (corresponding to the intracellular concentration of this ion) [22] along with 250 µM Mn^2+^. The presence of manganese ions is necessary for the activation of Pol ι [18,19]. The Pol ι enzymatic activity was estimated by the appearance of an additional specific band on electropherograms, which corresponds to the 18-mer product with misincorporated G opposite template T. Only Pol ι is capable of generating significant amounts of this product, since its formation is inhibited by Pol ι-specific aptamer [26]. Relying on this unique feature we have developed a method for the detection of Pol ι activity in cell extracts, named misGvA (misincorporation of G *versus* A) [24,25,27,28].

The DNA synthesis in cell extracts can yield three primary products (Figure 1A). Product I is synthesized as a result of the activity of correct DNA polymerases and contains only complementary nucleotides. Product II is synthesized by Pol ι and contains an incorrectly-incorporated G opposite template T. DNA polymerases in extracts of most mammalian tissues are unable to continue DNA synthesis bypassing the nucleotide misincorporated by Pol ι (the phenomenon called T-stop) [17,24]. The only exceptions to this rule are DNA polymerase(s) from testicular and tumor tissues [19,24]. In these latter cases the product II can serve as a substrate for further DNA synthesis, leading to the generation of product III. In this context, it is unclear whether Pol ι has any role in the synthesis of product III.

The acquired electropherogram demonstrates that solely product I is generated in cell extracts of transgenic loach larvae expressing inactive human Pol ι (negative control) (Figure 1B). At the same time, considerable amounts of product II, bearing misincorporated G opposite template T, are generated in loach larvae expressing the active form of Pol ι (Figure 1B,C). Additional bands in the range corresponding to 19-mer and 21-mer products can be also observed. The G/T mismatch induced by Pol ι is significantly restricted to downstream of T/A, which is confirmed by electrophoresis of the corresponding synthetic labeled oligonucleotides [29]. This indicates a capacity to continue further DNA synthesis using a template with aberrantly-incorporated G opposite to T (product III) (a capacity to bypass the T-stop). We have previously observed such phenomenon only in the extracts of mammalian tumor cells and testis [19,24].

### 3.2. The Role of Pol ι in the Bypassing of T-Stop

**Figure 2:**
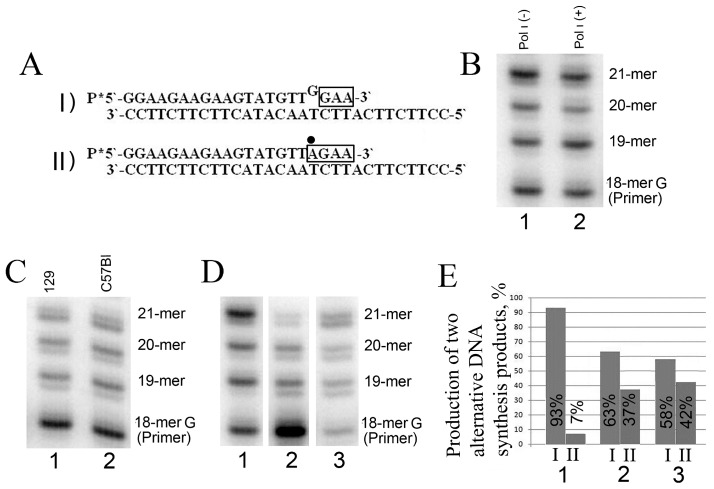
DNA synthesis in cell extracts using a substrate that contains a non-complementary nucleotide at the 3’-end of the primer. In all cases, quantitative parameters were determined from results of three to five independent experiments. (**A**) Alternative DNA synthesis products, generated in cell extracts with substrate No. 2. The frame highlights the nucleotides that are incorporated into substrate No. 2 during DNA synthesis. The dot above indicates the correct nucleotide incorporated in place of a mismatching nucleotide that was excised by exonucleases; (**B**) Electropherogram of DNA synthesis products generated in cell extracts of transgenic loach larvae that express either inactive (lane 1) or active Pol ι (lane 2) using substrate No. 2; (**C**) Electropherogram of DNA synthesis products that were generated in cell extracts of mice of the 129 line (lane 1) and the C57Bl line (lane 2); (**D**) Electropherogram of DNA synthesis products that were generated in cell extracts of loach larvae (lane 1) or in extracts of brain (lane 2) or testicular (lane 3) cells of adult fish; (**E**) Histogram representing the proportion of two alternative DNA synthesis products (sum of 18–21-mer) generated in tissue extracts of loach larvae and adult loaches using substrate No. 2 (the data was derived from electropherogram (D) and calculations were performed using ImageQuant software). Standard deviation in all experiments was within 5%.

To reveal the role of Pol ι in the bypassing of the T-stop, we have performed a primer extension reaction using substrate No. 2, which contained a non-complementary nucleotide at the 3’-end of the 18-mer primer. The electropherogram (Figure 2B) shows that the DNA synthesis on substrate No. 2 was equally efficient in cell extracts of loach larvae with both active and inactive forms of human Pol ι. Despite the presence of the active form of human Pol ι and the conditions optimized for its activity, the quantity of DNA synthesis products generated as a result of bypassing the T-stop was identical in both cases. These results indicate that Pol ι does not participate in further DNA synthesis after it incorporates an inappropriate nucleotide. 

According to the literature data, in certain cases, the participation of at least two DNA polymerases is essential to bypass damaged or modified nucleotides in the DNA strand [30,31]. Therefore, it is possible that Pol ι can interact with other DNA polymerases and thus promote DNA synthesis on substrates that incorporate non-complementary nucleotides. In order to definitively establish the role of Pol ι in bypassing the T-stop, we have performed additional experiments with cell extracts of mice of the C57Bl line (that possess the active Pol ι), and with cell extracts of 129 line mice (containing a nonsense codon mutation in the gene coding Pol ι) [32]. The extracts for these experiments were obtained from the testis, since this tissue has the highest level of Pol ι activity [25]. As can be seen in Figure 2C, DNA synthesis using substrate No. 2 was equal in both cases. Thus, it can be concluded that Pol ι does not participate in translesion DNA synthesis over the misincorporated nucleotide, and it is not related to bypassing the T-stop.

### 3.3. Developmental Changes in Corrective DNA Repair of Substrate Bearing Non-Complementary Nucleotide at the 3’-End

The synthesis of DNA using substrate No. 2, corresponding to the aberrant product generated by Pol ι on substrate No. 1, can proceed via two main routes. In the first route, the product with complementary nucleotides after unpaired G opposite to T is generated (product I at Figure 2A). In the second route, the incorporation of new nucleotides proceeds only after exonuclease excision of mismatching G, followed by incorporation of complementary nucleotides (product II in Figure 2A). The analysis of electrophoretically-separated products of DNA synthesis in loach larvae extracts using substrate No. 2 has demonstrated that the excision of unpaired G opposing T of template is not observed, and mainly product I is formed (more than 93%) (Figure 2D,E). At the same time, the analysis of electropherograms of DNA synthesis products with substrate No. 2 in cell extracts of different tissues (brain and testis) of adult loaches has demonstrated (Figure 2D,E) that about 40% of generated products undergo corrective excision of unpaired G opposite template T. 

Therefore, in loach larvae, the capacity to repair the inappropriately-incorporated nucleotide is reduced 5–6 fold in comparison with adult loach tissues. It is important to note that both tissues that contain the actively dividing cells (testis) and tissues that contain non-dividing cells (brain) from adult loaches were utilized in our experiments. Furthermore, it can be seen in Figure 2D,E that synthesis efficiency after an unpaired nucleotide is considerably higher in cell extracts of loach larvae (Figure 2D, lane 1) than in extracts of adult fish (Figure 2D, lanes 2 and 3). It is evident that a decreased DNA repair activity, along with the elevated activity of DNA polymerase(s) capable of bypassing the T-stop, is observed in loach larvae in comparison to adult loaches. 

The present study utilizes the previously-developed method (the misGvA approach) for the determination of Pol ι activity in extracts. This method is based on the difference in electrophoretic mobility between the products that contain correct and misincorporated nucleotides [25,28]. The advantage of this method is that it allows one to estimate DNA synthesis in cell extracts, which occurs as a result of a combination of various intracellular factors. To a certain extent, this method complements the results of the traditional experiments. Thus, it has been shown previously using RT-PCR and Western-blot that human breast cancer cells contain an increased amount of mRNA, which codes Pol ι and, therefore, an increased level of this protein, which explains the increased rate of mutagenesis [33]. On the other hand, we have shown using the misGvA method that cellular extracts of some of the malignant human cells are not only characterized by a higher activity of Pol ι, but also an ability to continue DNA synthesis after an incorrect nucleotide is inserted by this enzyme (a so-called phenomenon of overcoming the T-stop) [24]. One of the key results of the present work is that the T-stop is being overcome in cellular extracts of loach cells without Pol ι activity. 

## 4. Conclusions

Systematic studies of DNA synthesis, damage, and repair pathways using adult or embryonic fish have not been extensively reported. We have shown that cellular extracts of loach larvae embryos, in comparison to those of adult fish, have a significantly lower ability to remove the incorrect nucleotide in the process of DNA synthesis and replace it with the correct one. It is important to note that assaying DNA synthesis and repair in cellular extracts allows for the assessment of the integral activity of enzymes involved in the metabolism of DNA. Our results are in agreement with the data of other investigations, which demonstrated that the process of embryogenesis in fish involves a switch between several processes of DNA repair [34,35].

We assume that the early stages of embryogenesis require fast DNA synthesis, therefore the stability of the DNA structure becomes a less important factor. In contrast, for an adult organism, the genome’s stability is essential for long-term existence, and can be ensured by a set of complicated repair mechanisms. This hypothesis conforms with our long-term observations that 5% of loach larvae hatched under normal conditions have various growth anomalies. This feature may have evolutionary importance, as some of the mutations occurring at the early stages of an organism’s development may affect its ability to adapt to the environment. 

Considering the obtained data, it can be assumed that a character of synthesis and repair of DNA does not remain constant during fish development. It appears that the initial stages of development are characterized by more intensive DNA synthesis, while in terminal stages the repair activities become more prominent. The misGvA approach clearly indicates substantial changes in the DNA synthesis intensity, although the role of particular replicative and repair DNA polymerases in this process remains unclear and requires further study.
